# Structural pathways from emotional intelligence to psychological well-being: evidence of gender differences among Chinese young adults

**DOI:** 10.3389/fpsyg.2026.1811687

**Published:** 2026-04-23

**Authors:** Zelong Cao, Xia Kang, Yajun Wu

**Affiliations:** 1 Melbourne School of Population and Global Health, The University of Melbourne, Melbourne, VIC, Australia; 2 School of Mathematics and Information Science, Guangzhou University, Guangzhou, China; 3 School of Humanities, Foshan University, Foshan, China

**Keywords:** emotional intelligence, gender, psychological well-being, resilience, self-efficacy

## Abstract

**Background:**

Emotional intelligence (EI) has been linked to psychological well-being (PWB), but the mechanisms underlying this association and possible gender differences remain unclear among Chinese young adults. This study aimed to examine whether academic self-efficacy and academic resilience mediate the associations between four EI dimensions and PWB, and whether these structural pathways differ by gender.

**Methods:**

Using stratified random sampling, 951 Chinese undergraduates from three comprehensive universities in Guangzhou (M*_age_* = 19.52, SD = 0.84) completed validated self-report measures of EI, PWB, academic self-efficacy, and academic resilience. Structural equation modeling (SEM) and multi-group SEM were employed to estimate direct and indirect effects.

**Results:**

Academic self-efficacy significantly mediated the associations of the action-oriented EI dimensions (i.e., use of emotion and regulation of emotion) with PWB in both male and female groups. By contrast, academic resilience showed no significant indirect effects. In the structural model, others’ emotion appraisal was positively associated with academic resilience and PWB among females, but these associations were not significant among males.

**Conclusion:**

These findings identify academic self-efficacy as a key pathway linking EI to PWB in Chinese young adults, with a broadly consistent pattern across genders. Additionally, a gender-specific role for others’ emotion appraisal was identified. The results have implications for theory and for the development of well-being interventions for young adults.

## 1 Introduction

College students today are subject to a range of internal and external stressors, notably academic pressure, interpersonal challenges, and uncertainty regarding future career trajectories, with potential adverse implications for their PWB (Ryan and Baik, 2026). Educating for youth well-being is a core principle of higher education and a salient value orientation (Páez Gallego et al., 2020). Advancing this agenda requires identifying psychological determinants of young adults’ PWB. Among the many determinants of well-being, emotional intelligence has been recognized as a central construct in positive psychology, and an expanding literature links EI to health, quality of life, career adaptability and PWB (Carmeli et al., 2009; Baudry et al., 2018; Pong and Leung, 2023; Lomas et al., 2025). EI may be understood as an individual psychological resource that supports adaptive functioning and, in turn, higher levels of PWB (Lomas et al., 2025). However, the psychological mechanisms underlying this relationship remain underexplored (Ye et al., 2024). Drawing on emotional intelligence theory (Mayer et al., 2004) and psychological well-being theory (Ryff, 1989), EI, as a relatively stable personal trait, may facilitate the development of internal psychological resources such as self-efficacy and resilience, which are themselves positively associated with PWB (Costa et al., 2013; Akbari and Khormaiee, 2015; Buenconsejo et al., 2024). Given the importance of young adults’ PWB, it is vital to clarify the key precursors and the pathways by which it can be strengthened. Accordingly, the present study aims to determine whether EI is positively associated with young adults’ PWB and, if so, whether self-efficacy and resilience mediate this association.

Furthermore, existing studies have reported gender-related variation in EI and PWB (Fernández-Berrocal et al., 2012; Matud et al., 2022), and have examined whether gender moderates the association between the EI-PWB association (Brackett and Mayer, 2003; Schutte et al., 2007). However, the literature remains inconclusive, with findings that vary across studies (Urbón et al., 2025). Therefore, in addition to investigating the relationship between EI and PWB and its mediating pathways, it is essential to establish whether these associations are consistent across genders. As a response to this gap, the present study draws on a sample of Chinese undergraduates to empirically test the “EI → PWB” model and to further evaluate gender differences in the model’s structural relations. This study offers a more explicit account of the mechanisms through which young adults’ EI is associated with PWB. In addition, we extend prior research by examining gender differences in the model’s structural pathways. Taken together, these findings provide a theoretically grounded basis for developing and refining interventions designed to enhance PWB in young adults.

## 2 Literature review

### 2.1 Emotional intelligence theory and psychological well-being theory

Emotional intelligence is a cornerstone construct in emotional intelligence theory (Salovey and Mayer, 1990). Within this framework, EI is typically conceptualized as comprising three interrelated capacities: (a) the appraisal and expression of emotions in oneself and others, which entails the accurate and timely recognition of emotions in both self and others, coupled with the capacity for empathy; (b) the regulation of emotions in oneself and others, involving the ability to modulate one’s own emotional states, provide comfort or inspiration to others, and avert sustained engagement with negative emotions; and (c) the strategic use of emotions, wherein individuals leverage emotions to enhance cognitive processes and address challenges effectively (Salovey and Mayer, 1990; Mayer et al., 2004; Goleman, 2009). Given the pivotal role of emotion in psychological well-being (Adler and Hershfield, 2012), emotional intelligence theory offers a coherent account of the ways in which EI may contribute to enhanced PWB (Guerra-Bustamante et al., 2019).

The psychological well-being theory (Ryff, 1989, 2018) distinguishes PWB from subjective well-being across multiple dimensions, including philosophical origins, psychological underpinnings, focal points, conceptual frameworks, and assessment approaches. This theory underscores the distinct composition and framework of PWB, while also exploring the factors that precede or contribute to PWB (Ryff and Keyes, 1995; Huppert, 2009; Galderisi et al., 2015). Within this theoretical framework, a variety of factors contributing to PWB have been recognized, such as EI (Guerra-Bustamante et al., 2019), self-efficacy (Costa et al., 2013), and resilience (Avey et al., 2023). Accordingly, the present study draws on both emotional intelligence theory and psychological well-being theory to articulate the linkage between EI and PWB and to deepen conceptual understanding of these two constructs. In addition, positive psychology emphasizes positive subjective experience, positive individual traits, and supportive contexts that promote flourishing and optimal functioning (Seligman and Csikszentmihalyi, 2000). From this perspective, EI may be understood as a positive personal resource that supports adaptive functioning and contributes to higher PWB.

### 2.2 Links between EI and PWB

Within the framework of emotional intelligence theory, EI refers to the skills to perceive, understand, manage, and regulate emotions in oneself and in others (Salovey and Mayer, 1990). Individuals with higher EI typically demonstrate a stronger ability to accurately identify emotions, use emotional information to guide thinking, comprehend emotional dynamics, and regulate emotional responses (Mayer et al., 2008). Such competencies are typically associated with more adaptive functioning (e.g., greater stress tolerance and more effective problem solving) and more favorable life outcomes (e.g., better physical and mental health, higher-quality interpersonal relationships, and enhanced PWB) (Van Rooy and Viswesvaran, 2004). Conceptually, EI is often positioned as a key component of social intelligence, serving as a crucial interface between cognitive intelligence and the study of emotion (Mestre et al., 2016). Since the 1990s, EI has attracted sustained attention in both scholarly and managerial contexts. In some accounts, EI has been considered as consequential as intelligence quotient for explaining individual performance and success (Goleman, 2009) and individuals’ PWB (Bar-On and Parker, 2000).

Beyond theoretical accounts, the association between EI and PWB is also supported by a growing body of empirical evidence. For instance, a study of 646 Spanish adolescents reported that PWB was positively related to stronger abilities to understand and regulate emotions (Guerra-Bustamante et al., 2019). Using an experimental design, Ruiz-Aranda et al. (2012) further demonstrated that participation in an EI training program significantly improved adolescents’ capacity to understand, facilitate, and manage emotions, which in turn contributed to more favorable mental health outcomes. Similarly, a study of Year 8 students in Australia found that EI was associated with fewer negative emotional experiences and with higher levels of well-being (Lomas et al., 2025). Taken together, these studies provide convergent support for the positive association between EI and PWB across both correlational and intervention-based research. Despite this evidence base, notable gaps remain. First, much of the extant literature has focused predominantly on adolescent populations, with comparatively limited attention to adults. Second, empirical work has been conducted largely in Western cultural contexts, prompting the inquiry into whether the beneficial association between EI and PWB generalizes to Confucian heritage cultural settings.

### 2.3 Self-efficacy and resilience as mediators between EI and PWB

As the relationship between EI and PWB has been progressively elucidated, the mediating mechanisms underpinning this relationship have attracted increasing scholarly attention. For instance, a study of male college students in Lithuania found that social support partially mediated the connection between EI and PWB (Malinauskas, 2020). Similarly, research involving unemployed adults in Spain indicated that health-enhancing behaviors partially mediated the linkage between EI and well-being (Peláez-Fernández et al., 2022). In addition, a study of Chinese postgraduate students reported that social support and psychological resilience, along with their chain mediation effect, served as mediators linking EI and PWB (Zhang et al., 2022). Taken together, these findings suggest that EI may exert its influence on PWB through multiple intermediary processes. However, existing studies have tended to emphasize social and contextual mediators, while comparatively less attention has been devoted to psychological traits, such as self-efficacy and resilience (Costa et al., 2013).

Bandura’s social cognitive theory provides a particularly framework for understanding the mediating role of academic self-efficacy. Social cognitive theory emphasizes human agency, self-regulation, and beliefs about one’s capabilities as important determinants of motivation and functioning (Bandura, 1999). In academic contexts, students with higher EI may be better able to manage anxiety, frustration, and self-doubt, thereby strengthening their confidence in their capacity to meet academic demands successfully.

Academic self-efficacy, defined as an individual’s beliefs in their capability to organize and execute the actions required to succeed in academic tasks, may constitute an important psychological resource through which EI shapes adjustment and well-being. Empirically, existing research has established positive links between EI and self-efficacy (Di Fabio and Palazzeschi, 2008), as well as between self-efficacy and PWB (Costa et al., 2013), providing indirect support for the “EI → self-efficacy → PWB” mediational pathway. Thus, social cognitive theory and prior empirical findings together suggest that academic self-efficacy may be a key explanatory mechanism linking EI to PWB.

Fredrickson’s broaden-and-build theory provides an additional rational for the role of academic resilience. This theory proposes that positive emotions broaden individuals’ momentary thought-action repertoires and, over time, help build enduring psychological resources (Fredrickson, 2001). Although positive emotions were not directly measured in the present study, this perspective suggests that emotion-related capacities may facilitate the development of adaptive psychological resources such as resilience.

Resilience can be defined as an individual’s ability to adapt successfully and recover in the face of stress and adversity (Burton et al., 2010). In educational contexts, academic resilience reflects adaptive responses to academic pressure, setbacks, and study-related stressors. As an important psychological resource, resilience has been empirically linked to PWB (Smith and Hollinger-Smith, 2015; Guerra-Bustamante et al., 2019). Meanwhile, some studies have posited EI as an antecedent of resilience (Armstrong et al., 2011; Di Fabio and Saklofske, 2018). Accordingly, broaden-and-build theory, together with prior empirical evidence, supports the plausibility that academic resilience may mediate the relationship between EI and PWB.

Taken together, positive psychology provides a broader strengths-based context for the present model, social cognitive theory offers a clear rationale for the mediating role of academic self-efficacy, and broaden-and-build theory provides an additional basis for considering academic resilience as a potential resource-building pathway. On this basis, this study posited that both resilience and self-efficacy may mediate the association between EI and PWB. Given that prior studies on the “EI → PWB” relationship have typically reported partial mediation (Malinauskas, 2020; Peláez-Fernández et al., 2022; Zhang et al., 2022), the present study sought to further clarify the mechanisms through which EI influences PWB by testing a multiple-mediation model of “EI → resilience/self-efficacy → PWB.”

### 2.4 Gender difference in EI and PWB and their association

Drawing from the discussion on the wide-ranging benefits of EI, it is vital to further investigate potential variations in EI across different demographic groups, including gender, since gender could influence both EI, PWB, and related psychological characteristics such as self-efficacy and resilience. For example, women’s EI generally exceeds that of men, as evidenced by their greater superiority in emotional processing (Fernández-Berrocal et al., 2012), superior understanding and regulation of their own emotions (Ciarrochi et al., 2005; D’Amico and Geraci, 2022), and heightened sensitivity to the emotions of others (Hall and Mast, 2008).

Gender differences are also significantly reflected in PWB (Salk et al., 2017; Campbell et al., 2021; Zhu et al., 2025). A meta-analysis conducted by Salk et al. revealed that women reported lower levels of PWB compared to their male counterparts, experiencing higher levels of ill-being (Salk et al., 2017), including increased anxiety, depression, and frustration (Blanchflower and Bryson, 2024). In contrast, in a study involving adolescents aged 15 from 78 countries, Campbell et al. discovered that male adolescents reported significantly higher life satisfaction compared to female adolescents (Campbell et al., 2021), while also experiencing lower psychological distress, indicating a relatively higher level of PWB. Interestingly, although women report elevated levels of negative affect and exhibit higher incidences of depression, sadness, and loneliness, they paradoxically express greater happiness and life satisfaction compared to men (Blanchflower and Bryson, 2024). Such variability could be explained by cultural influences, disparities in measurement tools employed, and the inclusion or exclusion of age and socioeconomic status as covariates.

EI demonstrably affects PWB, with the nature and magnitude of this effect varying between genders. For example, women typically reported higher PWB, presumably owing to their superior ability in emotional regulation, which enables them to navigate emotional and social challenges with greater efficacy (Urbón et al., 2025). In brief, EI and PWB, along with their interrelations, show gender disparities. Yet, studies on the mediating effects of self-efficacy and resilience in this dynamic are limited, and analyses of gender differences in these proposed mediation modes are conspicuously absent.

### 2.5 The present research

**Figure 1:**
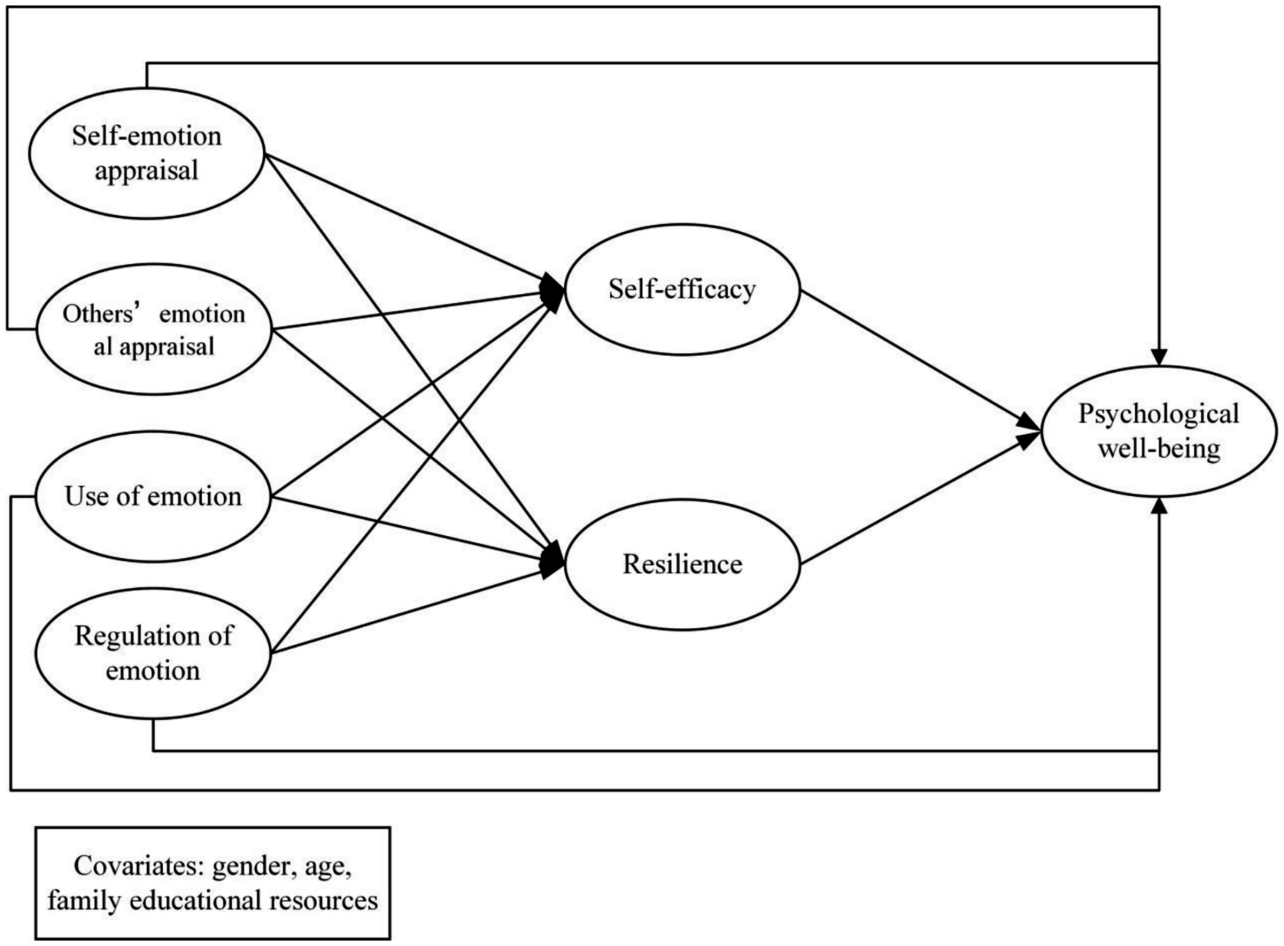
The proposed mediation model.

Drawing on the literature reviewed above, the present study is guided by one overarching question: how do the four dimensions of EI relate to PWB among Chinese undergraduates, through what roles do academic self-efficacy and academic resilience help explain these associations (Figure 1), and do these direct and indirect pathways differ by gender? To answer this question, we tested a gender-comparative multiple-mediation model in which academic self-efficacy and academic resilience were specified as mediators between the four EI dimensions and PWB. Based on this model, the following hypotheses were proposed.

*H1*: Each of the four EI dimensions is positively associated with academic self-efficacy, academic resilience, and PWB.

*H2*: Academic self-efficacy mediates the relationship between each of the four EI dimensions and PWB.

*H3*: Academic resilience mediates the association between each of the four EI dimensions and PWB.

*H4*: Gender moderates one or more structural paths in the mediation model (i.e., the associations among EI dimensions, academic self-efficacy, academic resilience, and PWB).

## 3 Methodology

### 3.1 Participants

The present study employed a cross-sectional, questionnaire-based correlational design to examine the direct and indirect associations between EI and PWB among Chinese undergraduates. To enhance representativeness and reduce sampling bias, stratified random sampling method was employed to recruit participants from three comprehensive universities in Guangzhou. More specifically, the strata were defined by the universities’ administrative affiliation, with one university selected from each category: a ministry-affiliated university, a provincially administered university, and a municipally administered university. The sample comprised 951 undergraduates aged 17–21 years (M*_age_* = 19.52, SD = 0.84), including 406 males (42.7%) and 545 females (57.3%). In terms of academic background, 55.8% of the participants were enrolled in science and engineering programs (e.g., mathematics and mechanical engineering), whereas 44.2% were majoring in humanities and social sciences (e.g., education, literature, and business administration). This distribution reflects a balanced representation of students from both STEM and non-STEM fields, ensuring that both groups were well represented in the sample.

To justify the adequacy of the sample size, a sensitivity power analysis was conducted using G*Power 3.1 (Faul et al., 2009). Using the F-test family (linear multiple regression: fixed model, *R*^2^ deviation from zero), with *α* = 0.05, desired power = 0.80, and seven predictors corresponding to the most complex structural equation in the model, the total sample of 951 was sufficient to detect a minimum effect size of *f*^2^ = 0.015. For the gender-specific analyses, the male (*n* = 406) and female (*n* = 545) subsamples were sufficient to detect minimum effect sizes of *f*^2^ = 0.036 and *f*^2^ = 0.027, respectively. These results indicate that the final sample was adequate for the planned analyses.

### 3.2 Study procedures, ethics and informed consent

Within China’s hierarchically stratified higher education system, comprehensive universities occupy the apex of the institutional hierarchy and offer broad disciplinary coverage spanning the humanities and social sciences, natural sciences, engineering and medicine (Shu et al., 2020). In contemporary China, these universities also function as pivotal institutional areas through which the state shapes younger cohorts by coupling high-level talent cultivation with ideological education and regulated pathways into the professional middle class (Mok and Wu, 2016; Xue and Li, 2021). We therefore used stratified random sampling to select three comprehensive universities within the Guangzhou Higher Education Mega Center and recruited undergraduate students from these institutions to complete a questionnaire survey. The questionnaire was administered in simplified Chinese, and the original English instruments were translated using a translation-back-translation protocol to ensure semantic equivalence with the source measures. A bilingual researcher first translated the Chinese version into English. The research team compared the original and back-translated versions and made minor wording revisions to improve semantic equivalence and readability in the study context. Ethical approval was obtained from the Human Research Ethics Committee of Guangzhou University (Reference No. [2023]133), and all participants provided written informed consent before data collection. Items were rated on a seven-point Likert scale, with higher scores indicating higher levels of the target construct.

### 3.3 Instruments

#### 3.3.1 Emotional intelligence scale

A 12-item short form adapted from the Emotional Intelligence scale (Wong and Law, 2002) was used to assess participants’ emotional intelligence. The scale comprises four subscales, each with three items. Representative items include “I have good understanding of my own emotions” (self-emotion appraisal), “I am a good observer of others’ emotions” (others’ emotion appraisal), “I would always encourage myself to try my best” (use of emotion), and “I am quite capable of controlling my own emotions” (regulation of emotion). The scale has been shown to possess strong psychometric properties in previous studies (Gong et al., 2019; Ye et al., 2024). In the present study, internal consistency reliability was acceptable for each subscale among male and female participants: self-emotion appraisal (*α* = 0.767 for males; α = 0.789 for females), others’ emotion appraisal (α = 0.799 for males; α = 0.763. female), use of emotion (α = 0.801 for males; α = 0.764 for females), and regulation of emotion (α = 0.867 for males; α = 0.866 for females).

#### 3.3.2 Psychological well-being scale

A six-item psychological well-being scale, adapted from Kim et al. (2020), was used to assess participants’ PWB. An example item is, “My life has a sense of direction or meaning to it.” In this study, Cronbach’s α for this scale ranged from 0.727 to 0.761, indicating good internal consistency.

#### 3.3.3 Academic self-efficacy scale

The eight items adapted from the MSLQ self-efficacy scale (Pintrich et al., 1991) were used to assess participants’ academic self-efficacy. One example item reads, “I expect to do well in school learning.” This scale has shown sound psychometric properties in samples of university students (Cordovani et al., 2024). In the present study, this scale showed good internal consistency in both male and female participants, with Cronbach’s α = 0.812 in each group.

#### 3.3.4 Academic resilience scale

Participants’ academic resilience was evaluated using the 6-item academic resilience scale, originally formulated by Martin and Marsh (2006). For example, one item on this scale is, “I do not let study stress get on top of me.” This scale has been widely utilized in prior research and has consistently exhibited strong psychometric properties (Martin, 2013; Liu et al., 2025). In the present study, the Cronbach’s α was 0.794 for the male group and 0.771 for the female group, reflecting satisfactory internal reliability across both groups.

Before testing the structural model, the measurement properties of the study measures were evaluated using CFA. Specifically, the internal consistency reliability was assessed using Cronbach’s alpha and composite reliability (CR). Convergent validity was evaluated using standardized factor loadings and average variance extracted (AVE) (see Table 1). Standardized loadings of at least 0.50, preferably 0.70 (Taber, 2018), CR values of at least 0.70 (Laborde et al., 2022), and AVE values of at least 0.50 (Hair et al., 2019) were taken as evidence of acceptable reliability and convergent validity. Discriminant validity was examined using the Fornell–Larcker criterion by comparing each construct’s AVE with its shared variance with other constructs. Although the AVE values for PWB and academic resilience were slightly below the recommended threshold of 0.50, they remain close to the criterion (both 0.45), and the corresponding CR values were above 0.70. Taken together, these results suggest that the convergent validity of the two constructs was considered acceptable (Cheung et al., 2024).

**Table 1:** AVE and reliability measures.

Construct	Factor loadings	CR	AVE	Discriminant validity						
				1	2	3	4	5	6	7
1. SEA	0.690–0.786	0.789	0.556	**0.746**						
2. OEA	0.700–0.756	0.780	0.542	0.419**	**0.736**					
3. UOE	0.688–0.812	0.788	0.555	0.392**	0.372**	**0.745**				
4. ROE	0.806–0.856	0.871	0.693	0.518**	0.371**	0.451**	**0.832**			
5. EFF	0.655–0.780	0.815	0.525	0.409**	0.345**	0.578**	0.620**	**0.725**		
6. RES	0.639–0.798	0.790	0.487	0.493**	0.402**	0.570**	0.589**	0.632**	**0.698**	
7. PWB	0.532–0.754	0.711	0.455	0.440**	0.405**	0.677**	0.577**	0.717**	0.624**	**0.675**

CR, Composite reliability. The bolded diagonal entries represent the square roots of the AVE, whereas the values below the diagonal are Pearson correlation coefficients. **p < 0.01.

### 3.4 Statistical analyses

Model fit for both the measurement and structural models was evaluated using the Chi-square statistic (*χ*^2^), the comparative fit index (CFI), the Tucker-Lewis index (TLI), the root mean square error of approximation (RMSEA), and the standardized root mean square error of approximation (SRMR). Consistent with conventional SEM reporting guidelines, CFI and TLI values of 0.90 or higher, together with RMSEA and SRMR values of 0.08 or lower, were considered indicative of acceptable model fit. More stringent criteria, namely CFI and TLI values of 0.95 or higher, RMSEA values of 0.06 or lower, and SRMR values of 0.08 or lower, were interpreted as reflecting good model fit (Hu and Bentler, 1999; Chen, 2007).

#### 3.4.1 Missing data

Prior to model estimation, the extent of missingness was assessed. Item-level missing data were minimal, with fewer than 1.1% of responses missing on any item. Little’s MCAR test was conducted to examine the missing-data mechanism, and the result was significant, *χ*^2^ (415) = 539.987, *p* < 0.001, indicating that the data were not missing completely at random (Little and Rubin, 2019). Accordingly, full-information maximum likelihood (FIML) was used to handle the missing data, following the default implementation in *M*plus (Muthén and Muthén, 2013).

#### 3.4.2 Measurement invariance

Before conducting multi-group analyses, measurement invariance across gender was evaluated to ensure that the latent constructs were comparable between male and female participants. Following established practice, configural, metric, and scalar invariance were tested sequentially (Shahzad et al., 2021). Invariance decisions were based on changes in model fit indices in addition to the chi-square (∆*χ*^2^), given the sensitivity of *χ*^2^ to sample size. Specifically, invariance was considered supported when deterioration in fit did not exceed ∆CFI ≥ −0.010, ∆RMSEA ≤ 0.015, ∆TLI ≤ 0.05, and ∆SRMR ≤ 0.030 for metric invariance or ∆SRMR ≤ 0.010 for scalar invariance (Chen, 2007; Putnick and Bornstein, 2016).

#### 3.4.3 Multi-group structural model

Then, a latent multi-group structural equation model was estimated to examine whether EI was associated with PWB directly and indirectly through academic self-efficacy and academic resilience, while controlling for age and family educational resources. To test whether structural path coefficients differed by gender, this study applied Wald *χ*^2^ test via the MODEL TEST command in *M*plus 8.3. When the omnibus Wald test was significant, we conducted follow-up tests by evaluating equality constraints for individual structural paths to identify specific gender differences; if no significant differences were detected, the follow-up testing was discontinued.

## 4 Results

### 4.1 Preliminary analyses

Table 2 reports the means, standard deviations, skewness, kurtosis, and Cronbach’s alpha coefficients for the study variables across gender groups. For all variables, skewness and kurtosis values fell within conventional thresholds for approximate univariate normality (|skewness|, |kurtosis|<3) (Tabachnick and Fidell, 2014), supporting approximate univariate normality of the observed variables. In addition, bivariate scatterplots of the study variables and standardized residual plots were inspected and showed no substantial departures from linearity. Furthermore, Cronbach’s coefficients (*α*) indicated satisfactory internal consistency of the scales.

**Table 2:** Descriptive analysis, normality indices and internal consistency reliability.

Variables	Male					Female				
	Mean	SD	S	K	α	Mean	SD	S	K	α
SEA	5.29	1.05	−0.77	0.74	0.767	5.11	1.09	−0.72	0.27	0.789
OEA	5.08	1.04	−0.58	0.52	0.799	5.12	0.93	−0.41	0.02	0.763
UOE	5.19	1.07	−0.70	0.81	0.801	5.20	0.95	−0.55	0.25	0.764
ROE	5.07	1.13	−0.78	0.57	0.867	4.68	1.16	−0.46	−0.07	0.866
EFF	4.76	1.07	−0.39	0.09	0.812	4.50	1.03	−0.22	−0.20	0.812
RES	5.12	1.00	−0.48	0.08	0.794	4.74	0.96	−0.09	−0.51	0.771
PWB	5.26	0.99	−0.44	−0.17	0.727	5.11	0.88	−0.41	0.34	0.761

SEA, self-emotion appraisal; OEA, others’ emotion appraisal; UOE, use of emotion; ROE, regulation of emotion; EFF, academic self-efficacy; RES, academic resilience; PWB, psychological well-being; S, skewness; K, kurtosis.

Table 3 presents the correlations among the main study variables for male university students (below the diagonal) and female university students (above the diagonal). Across both groups, the four EI dimensions were positively associated with academic self-efficacy, academic resilience, and PWB, and both academic self-efficacy and academic resilience were positively associated with PWB. Age was not correlated with any of the main study variables in either group and was therefore excluded from subsequent analyses. Among female young adults, family educational resources were positively correlated with others’ emotion appraisal, regulation of emotion, self-efficacy, resilience, and PWB, and negatively correlated with age. Among male young adults, family educational resources were positively correlated only with others’ emotion appraisal.

**Table 3:** Correlations matrix for the variables within male and female groups.

	1	2	3	4	5	6	7	8	9
1. SEA	–	0.389**	0.354**	0.457**	0.410**	0.441**	0.387**	0.016	0.022
2. OEA	0.467**	–	0.330**	0.361**	0.375**	0.420**	0.431**	0.012	0.174**
3. UOE	0.445**	0.416**	–	0.420**	0.594**	0.558**	0.640**	0.041	0.074
4. ROE	0.591**	0.405**	0.507**	–	0.604**	0.544**	0.520**	0.045	0.085*
5. EFF	0.395**	0.321**	0.570**	0.625**	–	0.637**	0.707**	0.014	0.139**
6. RES	0.550**	0.408**	0.610**	0.617**	0.610**	–	0.579**	0.045	0.118**
7. PWB	0.507**	0.379**	0.721**	0.654**	0.731**	0.686**	–	0.017	0.137**
8. Age	0.055	−0.044	0.038	0.033	0.033	0.034	0.077	–	−0.117**
9. FER	0.069	0.098*	0.000	0.025	0.081	0.048	0.063	−0.144**	–

Correlations for male young adults are reported below the diagonal; correlations for female young adults are reported above the diagonal. FER, Family educational resources. *p < 0.05; **p < 0.01; ***p < 0.001.

### 4.2 Measurement invariance across gender

Prior to analyzing differences in the mediation model between male and female groups, a multi-group CFA was initially conducted to assess measurement invariance (MI). MI across gender was examined to determine whether young men and women interpreted the four dimensions of EI, self-efficacy, resilience, and PWB in a comparable manner (Shahzad et al., 2021). As shown in Table 4, all model fit indices satisfied the commonly acceptable criteria for measurement invariance (Chen, 2007; Putnick and Bornstein, 2016). These findings confirm the MI of the measurement model, suggesting that factor loadings and intercepts are comparable between the male and female groups.

**Table 4:** Fit indices for measurement invariance tests across genders.

Model	*χ* ^2^	*df*	CFI (∆CFI)	TLI (∆TLI)	RMSEA (∆RMSEA)	SRMR (∆SRMR)
1. Configural	1210.203	418	0.923	0.907	0.063	0.045
2. Metric	1226.146	434	0.923 (0.000)	0.910 (0.003)	0.062 (−0.001)	0.049 (0.004)
3. Scalar	1342.112	457	0.914 (−0.009)	0.905 (−0.005)	0.064 (0.002)	0.057 (0.008)

### 4.3 Mediation analysis in the male group

**Figure 2:**
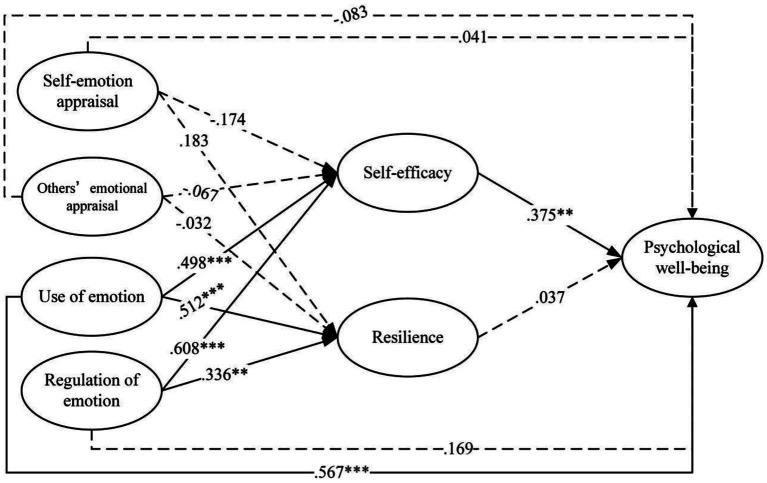
Mediation model in the male group. Family educational resources were included as a control variable. Dotted lines indicate paths involving the control variable and non-significant direct effects; solid lines indicate significant paths. **p < 0.01; ***p < 0.001.

The latent multi-group model, in which all parameters were freely estimated across groups, showed good fit to the data (*χ*^2^ (230) *=* 810.129, CFI = 0.945, TLI = 0.934, RMSEA = 0.052 and SRMR = 0.040). As shown in Figure 2, academic self-efficacy and academic resilience were examined as potential mediators linking EI to PWB among males. Specifically, use of emotion was positively associated with self-efficacy and resilience. Regulation of emotion was likewise positively connected with academic self-efficacy and academic resilience. By contrast, neither self-emotion appraisal nor others’ emotion appraisal was significantly related to academic self-efficacy or academic resilience. Academic self-efficacy, in turn, was positively associated with PWB, whereas academic resilience showed no significant association with PWB. The control variable of family educational resources was not significantly related to academic self-efficacy, academic resilience, or PWB.

Table 5 summarizes the direct and indirect effects of the four dimensions of EI on PWB in the male group. For the direct pathways, only use of emotion was positively associated with PWB; no significant direct effects were observed for self-emotion appraisal, others’ emotion appraisal or regulation of emotion. In the mediation analyses, use of emotion exhibited a significant indirect association with PWB via academic self-efficacy. In addition, regulation of emotion showed a significant indirect association with PWB through academic self-efficacy.

**Table 5:** Mediation model estimates for males.

Effects	Standardized β	SE	Bias-corrected CIs 95%	
			Lower	Upper
Indirect effect				
SEA → EFF → PWB	−0.065 n.s.	0.065	−0.223	0.005
SEA → RES → PWB	0.007 n.s.	0.046	−0.055	0.088
Direct effect	0.041 n.s.	0.115	−0.162	0.273
Indirect effect				
OEA → EFF → PWB	−0.025 n.s.	0.034	−0.104	0.032
OEA → RES → PWB	−0.001 n.s.	0.023	−0.048	0.016
Direct effect	−0.083 n.s.	0.073	−0.225	0.048
Indirect effect				
UOE → EFF → PWB	0.187*	0.076	0.062	0.353
UOE → RES → PWB	0.019 n.s.	0.111	−0.198	0.152
Direct effect	0.567***	0.161	0.314	0.895
Indirect effect				
ROE → EFF → PWB	0.228*	0.106	0.084	0.468
ROE → RES → PWB	0.012 n.s.	0.051	−0.088	0.111
Direct effect	0.169 n.s.	0.144	−0.106	0.445

n.s. *p* > 0.05; *p < 0.05; **p < 0.01; ***p < 0.001. SEA, Self-emotion appraisal; OEA, Others’ emotion appraisal; UOE, Use of emotion; ROE, Regulation of emotion; EFF, Self-efficacy; RES, Resilience; PWB, Psychological well-being.

### 4.4 Mediation analysis in the female group

**Figure 3:**
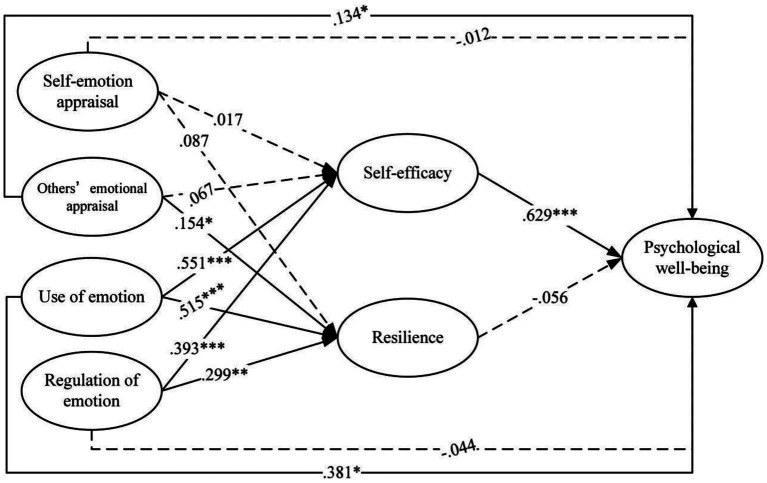
Mediation model in the female group. **p < 0.01; ***p < 0.001.

The mediating roles of academic self-efficacy and academic resilience in the relationships between EI and PWB are illustrated in Figure 3. Self-emotion appraisal was not significantly related to either academic self-efficacy or resilience. Others’ emotion appraisal was positively associated with resilience, but no with academic self-efficacy. In contrast, both use of emotion and regulation of emotion were positively associated with academic self-efficacy and resilience. Academic self-efficacy, in turn, was positively associated with PWB, whereas academic resilience showed no significant association with PWB. The control variable of family educational resources was not significantly related to academic self-efficacy, academic resilience, or PWB.

Table 6 summarizes the direct and indirect effects of EI on PWB among female students. For direct paths, others’ emotion appraisal and use of emotion were positively associated with PWB. By contrast, self-emotion appraisal and regulation of emotion showed no significant direct associations with PWB. In the mediation analyses, use of emotion exhibited a significant indirect association with PWB via academic self-efficacy. In addition, regulation of emotion was indirectly and positively associated with PWB through academic self-efficacy.

**Table 6:** Mediation model estimates for females.

Effects	Standardized β	SE	Bias-corrected CIs 95%	
			Lower	Upper
Indirect effect				
SEA → EFF → PWB	0.011 n.s.	0.045	−0.076	0.101
SEA → RES → PWB	−0.005 n.s.	0.019	−0.074	0.018
Direct effect	−0.012 n.s.	0.063	−0.132	0.115
Indirect effect				
OEA → EFF → PWB	0.042 n.s.	0.044	−0.030	0.145
OEA → RES → PWB	−0.009 n.s.	0.026	−0.074	0.037
Direct effect	0.134*	0.059	0.028	0.258
Indirect effect				
UOE → EFF → PWB	0.346***	0.081	0.206	0.526
UOE → RES → PWB	−0.029 n.s.	0.102	−0.268	0.093
Direct effect	0.381*	0.167	0.109	0.738
Indirect effect				
ROE → EFF → PWB	0.247***	0.067	0.126	0.393
ROE → RES → PWB	−0.017 n.s.	0.057	−0.151	0.057
Direct effect	−0.044 n.s.	0.103	−0.228	0.162

n.s. *p* > 0.05; *p < 0.05; ***p < 0.001. SEA, Self-emotion appraisal; OEA, Others’ emotion appraisal; UOE, Use of emotion; ROE, Regulation of emotion; EFF, Academic self-efficacy; RES, Academic resilience; PWB, Psychological well-being.

Finally, we examined whether the structural paths were equivalent between the male and female groups. Comparisons were restricted to paths that were statistically significant in the gender-specific models. The omnibus Wald test indicated significant cross-group differences in the set of paths (Wald *χ*^2^(5) = 11.250 and *p* < 0.05). Follow-up Wald tests showed that the paths from use of emotion to self-efficacy differed significantly between males and females (Wald *χ*^2^(1) = 6.908 and *p* < 0.01).

## 5 Discussion

Building on earlier work, the present study examined the structural relations among EI dimensions and tested whether academic self-efficacy and academic resilience mediate the association between EI and PWB across gender groups. Initially, we observed positive associations between all four EI dimensions and academic self-efficacy, academic resilience, and PWB, aligning with prior evidence that EI is linked to self-efficacy (Di Fabio and Palazzeschi, 2008; Wu et al., 2019), resilience (Schneider et al., 2013), and PWB (Carmeli et al., 2009; Lomas et al., 2025). Taken together, these findings suggest that interventions designed to enhance EI may also support young adults’ academic self-efficacy, academic resilience and PWB. From the perspective of emotional intelligence theory (Salovey and Mayer, 1990), individuals with high EI may be better able to perceive and manage their emotions effectively. These competencies may empower them to believe in their capabilities (academic self-efficacy), facilitate adaptive recovery from setbacks (academic resilience), and contribute to greater PWB.

Second, this study identified academic self-efficacy as a mediator of the association between EI and PWB, thereby confirming Hypothesis 2. This accords with previous research indicating that EI is positively related to self-efficacy and that self-efficacy, in turn, is associated with PWB (Costa et al., 2013). From the perspective of emotional intelligence theory, individuals who can accurately appraise their emotional responses and regulate negative emotions (e.g., anxiety or self-doubt) may be more likely to develop stronger confidence in their capacity to achieve success (Di Fabio and Palazzeschi, 2008; Wu et al., 2019). Greater academic self-efficacy, in turn, is linked to the adoption of more challenging goals and the use of more effective coping strategies when facing adversity, which may contribute to higher PWB (Roos et al., 2013; Kamil and AL-Hadrawi, 2022). Notably, academic self-efficacy significantly mediated the associations between the action-oriented EI dimensions (use of emotion and regulation of emotion) and PWB in both gender groups. At the same time, use of emotion also showed a significant direct association with PWB in both groups, indicating partial mediation for this pathway. These patterns suggest that academic self-efficacy is an important mechanism linking EI to PWB across genders, while some EI dimensions may also relate to PWB directly. One possible interpretation is that males may place greater emphasis on control and self-mastery than females (Schunk and Meece, 2006), rendering self-efficacy a particularly salient pathway through which EI is associated with PWB among males. Moreover, within a Confucian heritage cultural context, females may be more strongly socialized to express emotions and to seek interpersonal support, which could allow EI to relate to PWB both directly and indirectly. In contrast, males may rely more heavily on intrapersonal resources such as self-efficacy (Lu and Gilmour, 2004; Wong and Tsai, 2007).

Third, academic resilience did not emerge as a significant mediator of the EI-PWB association in either the male or female group. Therefore, Hypothesis 3 was not supported. Although EI was positively associated with academic resilience—consistent with previous evidence (Schneider et al., 2013; Magnano et al., 2016)—academic resilience did not account for the association between EI and PWB when considered alongside academic self-efficacy. Several explanations may be plausible. First, in a Confucian heritage cultural context, emotionally intelligent individuals may experience better PWB primarily through enhanced perceived competence, rather than through resilience alone (Ye et al., 2024). Second, given the substantial correlation between self-efficacy and resilience, modeling both pathways simultaneously may have meant that the indirect effect was largely captured by self-efficacy, leaving academic resilience with little unique mediating influence (Mackinnon, 2008). Third, prior work suggests that self-efficacy may influence well-being indirectly via resilience (Djourova et al., 2020), implying that self-efficacy may represent a more proximal pathway to PWB than resilience in this model.

In addition, the findings indicate both shared and gender-specific pathways linking EI to PWB. Notably, others’ emotion appraisal was directly and positively associated with PWB among females, whereas this pathway was not significant among males. This difference may reflect females’ greater interpersonal sensitivity and emotional expressiveness. Females are often socialized to attend more closely to relational dynamics and emotional cues (Hall and Mast, 2008; Barrett and Bliss-Moreau, 2009). Norms surrounding emotional openness and caregiving roles may further heighten the salience of accurately appraising others’ emotions for females’ well-being (Fischer et al., 2018). In contrast, males’ PWB may be less contingent on interpersonal emotional proficiencies (Trentini et al., 2022). More broadly, in both gender groups, use of emotion showed both a direct association with PWB and an indirect association via academic self-efficacy. Regulation of emotion showed a significant indirect association with PWB via academic self-efficacy, but its direct association with PWB was not significant. In contrast, self-emotion appraisal showed no significant effects, and the effects of others’ emotion appraisal were limited to a direct pathway among females. Taken together, these patterns suggest that actively deploying and regulating emotions may be more consequential for PWB than emotional awareness alone, potentially because such skills translate emotional understanding into adaptive action and stronger academic self-efficacy, which in turn supports well-being (Salovey and Mayer, 1990; Pauletto et al., 2021).

## 6 Theoretical and practical implications

The present study was primarily grounded in emotional intelligence theory and psychological well-being theory, while also drawing on complementary insights from positive psychology, social cognitive theory, and broaden-and-build theory. Together, these perspectives help explain why EI may function as a positive personal resource, why academic self-efficacy may serve as an important agentic mechanism, and why academic resilience may be conceptualized as a potential resource-building pathway linking EI to PWB. Additionally, the observed differential mediation effects between males and females suggest that gender dynamics influence the PWB of young adults. This finding aligns with the broader body of research on gender disparities in social development and social services, highlighting persistent inequalities and providing insights to guide the direction of future investigations.

Considering the established association between EI and PWB, it is imperative to prioritize the cultivation of emotional intelligence among young adults. Parents and educators play a critical role in the cultivation of EI in young adults. Their influence is exerted through the creation of a democratic, engaging, and supportive family atmosphere, alongside the provision of emotional and instrumental assistance in educational contexts (Szcześniak and Tułecka, 2020; Wan et al., 2023). Moreover, considering the mediating role of self-efficacy, strategies and interventions (e.g., health education program and intervention program) are suggested to be adopted to optimize self-efficacy in young adults (Van Dinther et al., 2011; Joseph et al., 2014), thereby enhancing their PWB. Additionally, the action-oriented dimensions of EI, in contrast to the purely cognitive understanding of self and others’ emotions, exert a significant influence on PWB. From a comparative perspective, females exhibit greater sensitivity in appraising others’ emotions compared to males, and this increased sensitivity exerts a substantial influence on PWB. Thus, significant others can create a conducive environment for the cultivation of interpersonal proficiencies across both genders.

## 7 Limitations and future directions

However, three limitations should be acknowledged. First, the study’s reliance on self-report measures may have introduced social desirability bias. Future work could strengthen measurement validity by integrating assessments from significant others, such as parents, teachers, or peers. Second, because the study was conducted within a Confucian heritage cultural context, the findings may not be readily generalizable to non-Confucian settings. Future research should therefore examine these associations across diverse cultural frameworks, including Western and Islamic contexts, to establish the robustness and boundary conditions of the observed relationships. Third, the cross-sectional design precludes causal inference. Future research could profit from incorporating longitudinal approaches or intervention-oriented research designs to clarify the directionality and causal mechanisms linking the study variables.

## 8 Conclusion

This preliminary study examined the association between EI and PWB and assessed gender differences in the structural relations among Chinese young adults shaped by Confucian heritage culture. Although exploratory in scope, the findings offer meaningful insights. First, all four EI dimensions were positively associated with academic self-efficacy, academic resilience, and PWB. Second, academic self-efficacy mediated the relationships between two EI dimensions (i.e., the use of emotion and the regulation of emotion) and PWB in both male and female groups. Third, academic resilience did not emerge as a significant mediator of the EI–PWB association. Fourth, others’ emotion appraisal was significantly and positively related to PWB among females, whereas this relation was not significant among males.

## Data Availability

The raw data supporting the conclusions of this article will be made available by the authors, without undue reservation.
